# iTRAQ-Based Quantitative Proteomic Analysis of Cotton Roots and Leaves Reveals Pathways Associated with Salt Stress

**DOI:** 10.1371/journal.pone.0148487

**Published:** 2016-02-03

**Authors:** Tingting Chen, Lei Zhang, Haihong Shang, Shaodong Liu, Jun Peng, Wankui Gong, Yuzhen Shi, Siping Zhang, Junwen Li, Juwu Gong, Qun Ge, Aiying Liu, Huijuan Ma, Xinhua Zhao, Youlu Yuan

**Affiliations:** State Key Laboratory of Cotton Biology, Institute of Cotton Research of CAAS, Anyang, 455000, P. R. China; Murdoch University, AUSTRALIA

## Abstract

Salinity is a major abiotic stress that affects plant growth and development. In this study, we performed a proteomic analysis of cotton roots and leaf tissue following exposure to saline stress. 611 and 1477 proteins were differentially expressed in the roots and leaves, respectively. In the roots, 259 (42%) proteins were up-regulated and 352 (58%) were down-regulated. In the leaves, 748 (51%) proteins were up-regulated and 729 (49%) were down-regulated. On the basis of Gene Ontology (GO) and Kyoto Encyclopedia of Genes and Genomes (KEGG) pathway enrichment analysis, we concluded that the phenylalanine metabolism and starch and sucrose metabolism were active for energy homeostasis to cope with salt stress in cotton roots. Moreover, photosynthesis, pyruvate metabolism, glycolysis / gluconeogenesis, carbon fixation in photosynthetic organisms and phenylalanine metabolism were inhabited to reduce energy consumption. Characterization of the signaling pathways will help elucidate the mechanism activated by cotton in response to salt stress.

## Introduction

Salinity is one of the major abiotic stresses that severely limit plant growth and development worldwide. It is estimated that more than 6% of the world’s total landmass and approximately 20% of irrigated landmass is affected by salinity[1]. Salt stress causes water deficit, ion toxicity, nutrient imbalance, and oxidative stress, leading to cellular damage and growth reduction, and even plant death [2–4].

Plant salinity tolerance is a complex phenomenon that involves physiological, biochemical, and molecular processes [1,5]. At the cellular level, plant roots function as the primary organ for sensing salinity so that they can respond rapidly to maintain functionality. Toxic ions are transported into the plant root along with the water stream, which moves from the soil to the vascular system of the root via several routes, including the symplastic and apoplastic pathways [2]. The ability of plants to maintain low cytosolic salt concentrations is due to their employment of selective ion uptake, ion exclusion[6] and compartmentalization; this is exemplified by the management of toxic levels of Na^+^ in vacuoles [7,8]. At the molecular level, several root proteins, such as plasma membrane and vacuolar H^+^-ATPase and Na^+^/H^+^ antiporters, play an essential role in ion uptake and transport [9,10]. In addition, antioxidant enzymes and soluble proteins in the cytoplasm have an important function in protecting cells from salt-induced oxidative stress[11,12].

Previously reported plant proteomics studies were limited to 2D gel electrophoresis analysis of roots [13,14]. However, the disadvantages of the 2D gel technique limited its application for comprehensive analysis of proteome changes [15–19]. Although most proteomics studies in crops have used 2D gel approaches [20], alternative methods are now available. Isobaric tags for relative and absolute quantitation (iTRAQ) is currently one of the most robust techniques that allows quantification of proteins on the basis of peptide labeling, as well as the identification and accurate quantification of proteins from multiple samples within broad dynamic ranges of protein abundance[21–24]. Moreover, some important post-translational modification information, such as proteolytic cleavage, glycosylation, or phosphorylation, may be retained. In addition, the proteome generated by iTRAQ typically consists of millions of peptides, from which substantial information on the proteins can be extracted with the use of bioinformatics tools.

Cotton (*Gossypium hirsutum* L.) produces an essential commodity, namely fibers for use in textiles, and cottonseed is a source of oil. Although it is classified as a salt-tolerant crop, this tolerance is actually limited, and varies according to the growth and developmental stages of the plant [25]. The effect of salinity on the germination, vegetative growth, and yield of cotton has been reported [26–28]. Breeders have sought to make cotton more tolerant to salt through various methods, including traditional plant breeding and biotechnological approaches such as creating transgenic cotton.

In this study, we used iTRAQ to assess proteome changes and identify proteins that were differentially expressed in cotton roots and leaves in response to NaCl concentration. On the basis of enrichment analysis of Gene Ontology (GO) annotations and Kyoto Encyclopedia of Genes and Genomes (KEGG), these differentially expressed proteins with functions in several biologically important pathways (phenylalanine metabolism and starch and sucrose metabolism for roots; photosynthesis, pyruvate metabolism, glycolysis / Gluconeogenesis, carbon fixation in photosynthetic organisms, and phenylalanine metabolism for leaves) we were likely important in the response to salt stress. Further characterization of these differentially expressed proteins will help to elucidate the signaling pathways activated by cotton in response to salt stress.

## Materials and Method

### Plant materials

Saline-tolerant seeds of *Gossypium hirsutum* cv. CCRI-79 were obtained from the National Medium-Term Gene Bank of Cotton in China and soaked in sterile deionized water at 28°C for 6 h. The method of seed planting was performed as described previously[29].

### Salt treatment

Plants were cultured under non-saline conditions for 10 d to ensure full establishment before starting the salinity treatments. Salt stress treatment was initiated by providing the plants with full-strength Hoagland’s solutions containing 0 or 240 mM NaCl. To avoid osmotic shock, salt concentrations were increased daily by 40 mM NaCl, until reaching the required concentration. Three experimental replicates were performed. After 1 week, the roots and leaves of cotton were frozen immediately in liquid nitrogen and stored at −80°C.

### Protein preparation and iTRAQ labeling

iTRAQ analysis was implemented at BGI(Shenzhen, China). Total proteins were extracted from the roots and leaves tissue of CCRI-79 plants using a phenol extraction procedure [30,31]. Two biological replicates were carried out for each sample.

Protein concentrations were determined using the Bradford method [32]. An equal amount of protein was prepared for each biological replicate. Protein samples from the roots and leaves were reduced with 10 mM DTT, alkylated with 55 mM iodoacetamide, digested using sequencing grade trypsin (Promega) at a ratio of 1:10 (w:w) for12 h at 37°C, and labeled using iTRAQ 8-plex kits (AB Sciex Inc., MA, USA) according to manufacturer’s manual. The root samples were labeled with iTRAQ tags 113 and114 (CK), 117 and 118 (salt treatment). The leaf samples were labeled with tags 115 and116 (CK), 119 and 120 (salt treatment).

### Strong cation exchange

After labeling and quenching, the samples of roots and leaves were combined and lyophilized, respectively. The peptide mixture was dissolved in 4 mL strong cation exchange (SCX) buffer A (25% v/v acetonitrile, 25 mMNaH_2_PO_4_, pH 2.7). The peptides were fractionated on a Shimadzu LC-20ABHPLC system (Shimadzu, Kyoto, Japan) using an Ultremex SCX column (4.6 × 250 mm). Peptides were eluted at a flow rate of 1 mL/min with elution buffer B (25% v/v acetonitrile, 25 mM NaH_2_PO_4_, 1 M KCl, pH 2.7). The absorbance at 214 nm was monitored and 20 fractions were collected. Samples of each fraction were dried and desalted before LC-ESI MS/MS analysis [33,34].

### LC-MS/MS analysis

The LC-MS/MS analysis was performed as described previously [33,34]. Peptides of each fraction (5-μL injections) were resolved insolvent A (5% acetonitrile, 0.1% formic acid) and centrifuged at 20,000 *g* for 10 min. The supernatant was separated using a Shimadzu LC-20AD Nano-HPLC system with a flow rate of 300 nL/min. Peptides were eluted by application of a linear gradient from 5% solvent B (95% acetonitrile v/v, 0.1% formic acid) to 35% solvent B over 35 min, followed by ramping up to 60% solvent B over 5 min, up to 80% in 2 min and maintained for 1 min; chromatographic conditions (5%) were restored in 1 min and equilibrated in solvent A for 10 min.

For the Triple TOF analysis, the AB SCIEX Triple TOF 5600 System (Concord, USA) was applied. Data were acquired using an ion spray voltage of 2.5 kV, curtain gas of 30 PSI, nebulizer gas of 15 PSI, and an interface heater temperature of 150°C. The MS was operated with a RP greater than or equal to 30,000 FWHM for TOFMS scans. For information-dependent data acquisition (IDA), survey scans were acquired in 250 ms and as many as 30 product ion scans were collected if exceeding a threshold of 120 counts per second (counts/s) and with a 2+ to 5+ charge-state. Total cycle time was fixed to 3.3 s. The Q2 transmission window was 100 Da for 100%. Four time bins were summed for each scan at a pulser frequency value of 11 kHz through monitoring of the 40 GHz multichannel TDC detector with four-anode/channel detection. A sweeping collision energy setting of 35 ± 5 eV, coupled with iTRAQ adjust rolling collision energy was applied to all precursor ions for collision-induced dissociation. Dynamic exclusion was set for 1/2 of peak width (18 s), and then the precursor was refreshed off the exclusion list. 5600 MS Converter was used to convert raw data files acquired from the Orbitrap into MGF files.

### iTRAQ protein identification and quantification

Protein identification and quantification was performed using the Mascot 2.3.02 search engine (Matrix Science, Boston, MA). The protein mass were predicted by website http://www.expasy.ch/tools/ based on the protein sequences. The following search settings were used as described previously [35,36]. Searches were made against our cotton_AD_nr database, including protein sequences from the *G*. *hirsutum* L. genome (AADD) [37]. The search parameters were as follows: threshold set-off at 0.05 in the ion-score cutoff (with 95% confidence); MS/MS fragment ion mass tolerance of ±0.1 Da; enzyme specificity was set to trypsin with one missed cleavage; peptide tolerance was set at 10 ppm; fixed modifications of carbamido methylation at Cys and iTRAQ 8 plex at Lys and the N-terminal amino group of peptides; variable modifications of oxidation at Met, iTRAQ 8 plex at Tyr, and glutamine as pyroglutamic acid; peptide change was set at Mr and monoisotopic mass was chosen; charge states of peptides were set to +2 and +3. Only peptides with significance scores greater than “identity_ score” were counted as identified. Considering that multiple MS/MS spectra match to one peptide, normalization of the signal intensities of each MS/MS spectra was performed to find the most likely expression ratio for a given peptide. To demonstrate the reproducibility of the replicates, protein abundances between various biological replicates were compared, and ratios for each protein in each comparison were normalized to 1. The difference was plotted against the percentage of the proteins quantified. For quantitative changes, a 1.2-fold cutoff was set to determine up-regulated and down-regulated proteins, with a p-value < 0.05 present in at least two replicates[34].

### Bioinformatic analysis of proteins

Functional category analysis was performed with Blast2GO software (http://www.geneontology.org)[38]. To take advantage of the current knowledge of biochemical pathways and other types of molecular interactions, we used KEGG databases (http://www.genome.jp/kegg/pathway.html)[39,40]. GO and pathway enrichment analysis were performed to determine which functional subcategories and metabolic pathways were overrepresented by the differentially accumulated proteins.

## Results

### Quantitative identification of cotton roots and leaves proteins using iTRAQ

**Fig 1:**
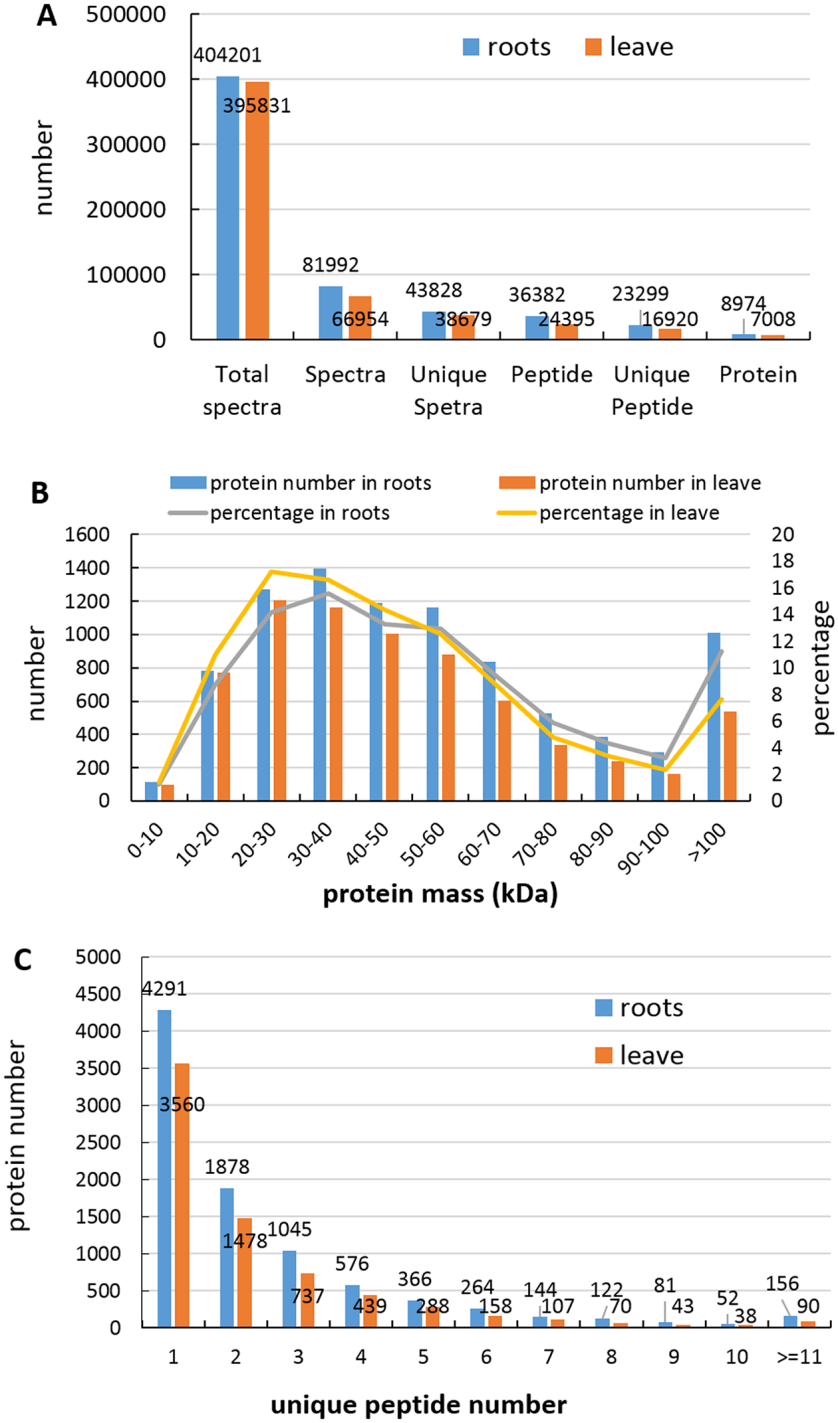
Identification and analysis of DEPs from roots and leaves in cotton. (A) Total spectra, spectra, unique spectra, peptides, unique peptide, and proteins identified from iTRAQ proteomic analysis. (B) Identified proteins were grouped according to the protein mass. (C) Number of peptides that match to proteins as indicated by MASCOT 2.3.02.

In previous study, it was found that salt stress significantly reduced the growth rates, surface area, volume, and average diameter of the cotton roots and the dry weights of roots and leaves in cotton variety CCRI-79. To monitor the dynamic changes, cotton variety CCRI-79 was treated with 0 and 240 mM NaCl, respectively. A salt-induced proteomic analysis by iTRAQ was performed in roots and leaves. After merging data from the two replicates, a total of 404201 and 395831 spectra were generated from roots and leaves. Ultimately, we obtained 43828 unique spectra, 36382 identified peptides including 23299 unique peptides and 8974 identified proteins from roots. Meanwhile, we obtained 38679 unique spectra, 24395 identified peptides including 16920 unique peptides and 7008 identified proteins from leaves (Fig 1A). The protein mass was normally distributed. In roots, the number of 10–50, 50–100 and above 100 kDa protein is 4758 (53%), 3206 (36%) and 1010 (11%), respectively. In leaves, the number of 10–50, 50–100 and above 100 kDa protein is 4247 (60%), 2225 (32%) and 536 (8%), respectively (Fig 1B). The distribution of peptide number is shown in Fig 1C. In roots, the proteins with a single peptide, 2–5 peptides, 6–10 peptides and above 11 peptides comprise 4191, 3865, 663 and 156, respectively. In leaves, the proteins with a single peptide, 2–5 peptides, 6–10 peptides and above 11 peptides comprise 3560, 2942, 416 and 90, respectively (Fig 1C).

### Functional categories of differentially expressed proteins

**Fig 2:**
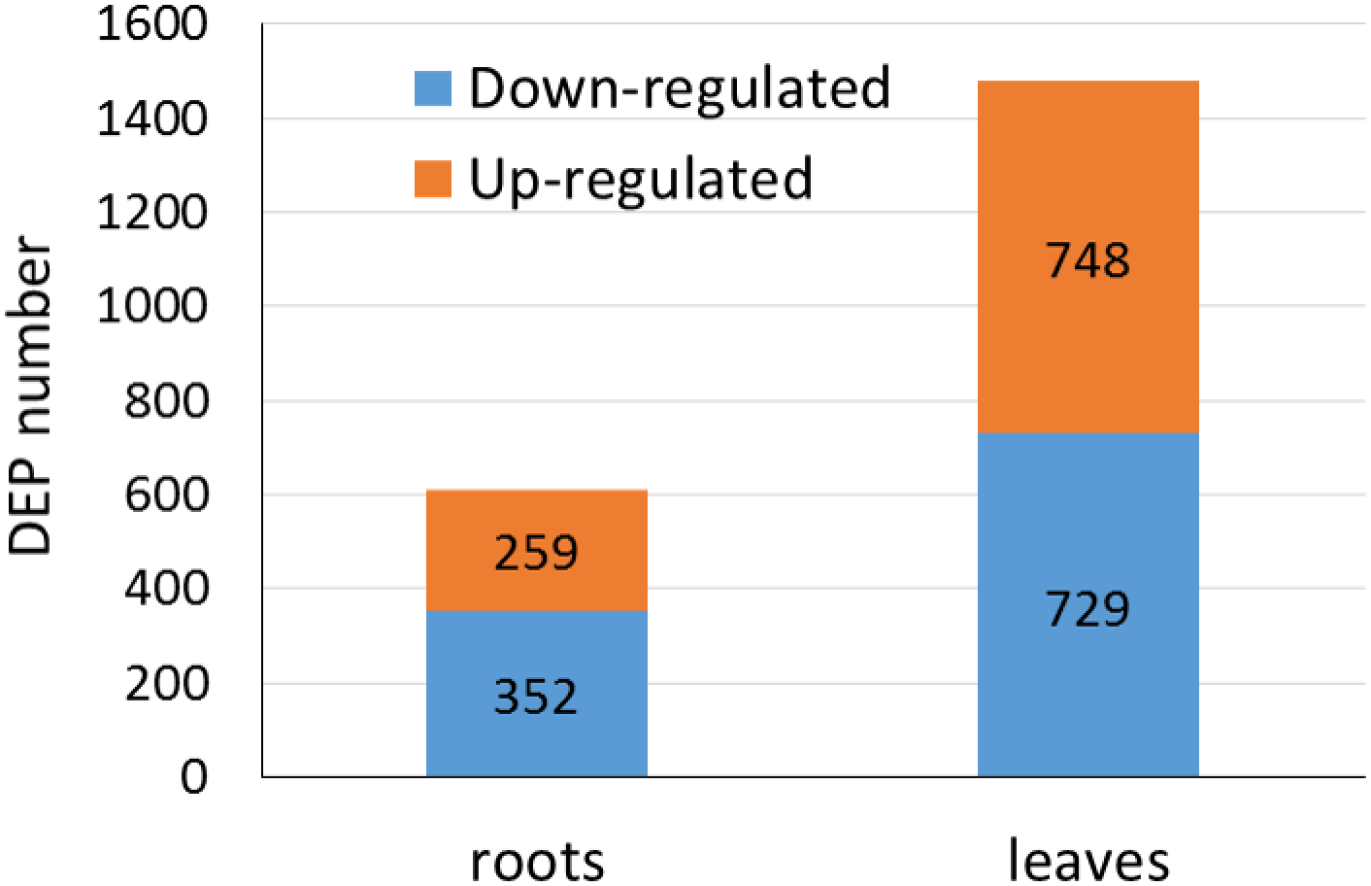
Number of up- or down-regulated proteins under salt stress in roots and leaves.

According to previous studies [15,41], differentially regulated proteins are defined based on a 1.2–1.5-fold change threshold. In this research, any proteins changed with ≥ 1.2-fold difference and a P value <0.05 would be designated as significant differently expression proteins (DEPs). Using these criteria, 611 and 1477 DEPs were identified in the roots and leaves, respectively (S1 Table). In the roots, 259(42%) proteins were up-regulated and 352 (58%) were down-regulated compared with the control. In the leaves, 748 (51%) proteins were up-regulated and 729 (49%) were down-regulated (Fig 2).

**Fig 3:**
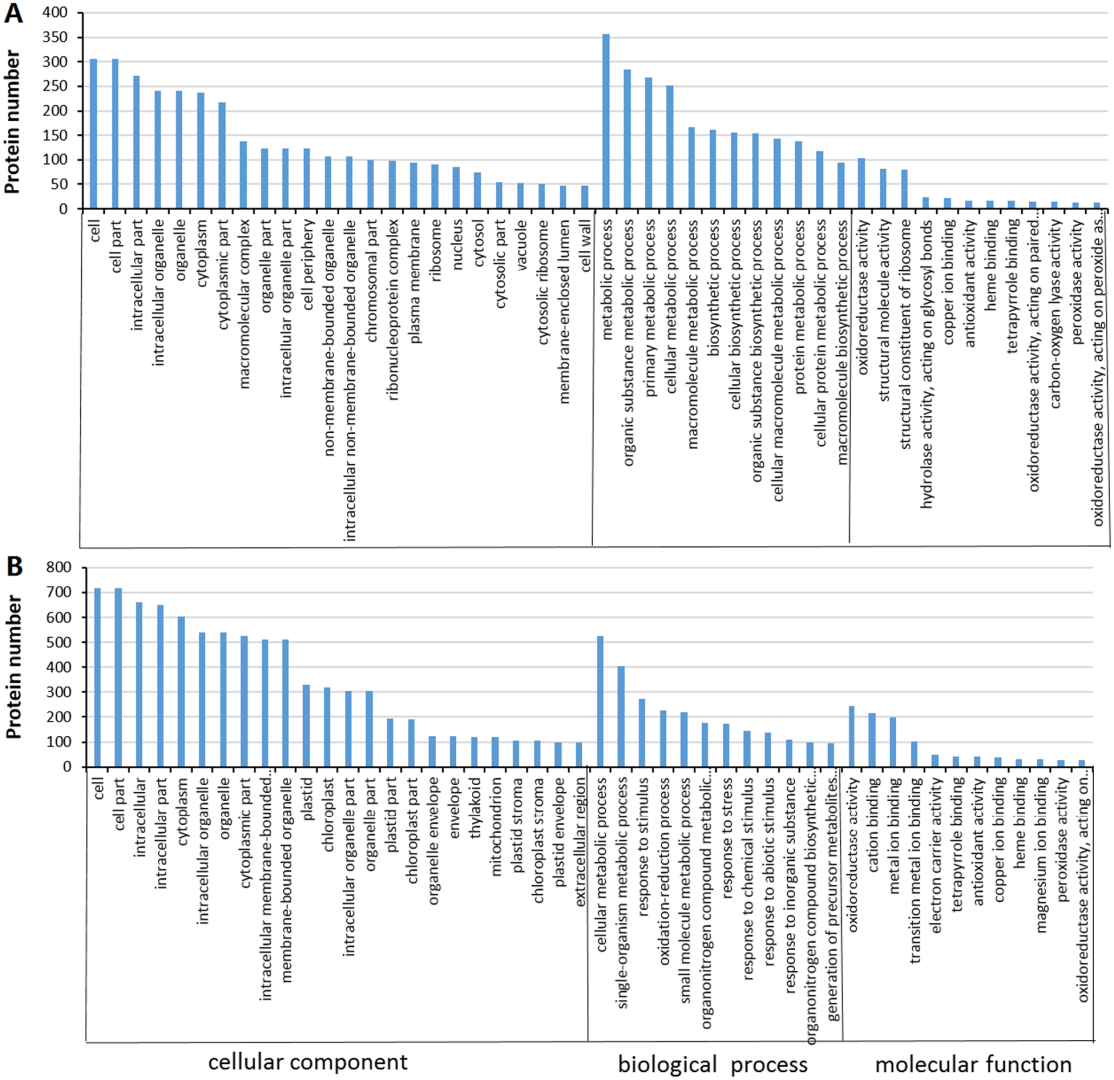
Gene Ontology (GO) classification of the DEPs in cotton. (A) GO classification of the DEPs in roots; (B) GO classification of the DEPs in leaves. The number of each category is displayed based on cellular components, molecular function, or biological process.

To gain insight into the functional categories that were altered following treatment with 240 mM NaCl, GO enrichment analysis of DEPs was conducted. The DEPs were classified into two groups, biological process and cellular component on the basis of GO enrichment analysis (Fig 3). In the roots, the main biological functional categories represented were metabolic process (356), organic substance metabolic process (284), primary metabolic process (268), and cellular metabolic process (251). For cellular components, proteins were predominantly distributed in the cell (306), cell part (306), intracellular part (271), intracellular organelle (241) and organelle (241)-related components (Fig 3A, S2 Table). In the leaves, the main biological functional categories were cellular metabolic process (527), single-organism metabolic process (404) and response to stimulus (274). According to the cellular components properties, these proteins were mainly classified into cell (716), cell part (716), intracellular (660) and intracellular part (649) (Fig 3B, S2 Table).

In order to analyze the metabolic pathways that responded to salt stress, 484 and 1100 DEPs were further investigated using the KEGG database in roots and leaves, respectively. These DEPs from roots were found to been enriched in biosynthesis of secondary metabolites (22.1%), ribosome (20.9%), phenylpropanoid biosynthesis (5.8%), phenylalanine metabolism (4.1%) and starch and sucrose metabolism (4.1%). The DEPs from leaves were found to been enriched in metabolic pathways (37.4%), biosynthesis of secondary metabolites (21.6%), ribosome (6.7%), photosynthesis (4.9), pyruvate metabolism (3.7%), glycolysis/Gluconeogenesis (3.7%), carbon fixation in photosynthetic organisms (3.4%), glutathione metabolism (3.0%) and phenylalanine metabolism (2.3%) (Table 1).

**Table 1:** Pathway enrichment analysis of DEPs.

Pathway	Proteins number	P value	Pathway ID
**roots**			
Biosynthesis of secondary metabolites	107	0.0123142	ko01110
Ribosome	101	3.27E-44	ko03010
Phenylpropanoid biosynthesis	28	0.000216818	ko00940
Phenylalanine metabolism	20	0.001024538	ko00360
Starch and sucrose metabolism	20	0.01059125	ko00500
Amino sugar and nucleotide sugar metabolism	17	0.02660386	ko00520
Phagosome	14	0.03063376	ko04145
Flavonoid biosynthesis	13	0.02118993	ko00941
Alanine, aspartate and glutamate metabolism	12	0.007096636	ko00250
Arginine and proline metabolism	11	0.0493542	ko00330
alpha-Linolenic acid metabolism	10	0.003776889	ko00592
Nitrogen metabolism	10	0.02299324	ko00910
Biosynthesis of unsaturated fatty acids	7	0.007946874	ko01040
Flavone and flavonol biosynthesis	7	0.03763566	ko00944
Sesquiterpenoid and triterpenoid biosynthesis	4	0.04798942	ko00909
**leaves**			
Metabolic pathways	411	7.80E-05	ko01100
Biosynthesis of secondary metabolites	238	0.007007947	ko01110
Ribosome	74	0.0177717	ko03010
Photosynthesis	54	4.39E-13	ko00195
Pyruvate metabolism	41	0.001799041	ko00620
Glycolysis / Gluconeogenesis	41	0.01159367	ko00010
Carbon fixation in photosynthetic organisms	37	0.00237416	ko00710
Glutathione metabolism	33	0.000339012	ko00480
Ascorbate and aldarate metabolism	28	0.03392843	ko00053
Phenylalanine metabolism	25	0.004105766	ko00360
Porphyrin and chlorophyll metabolism	22	0.0011575	ko00860
Aminoacyl-tRNA biosynthesis	20	0.0169819	ko00970
Propanoate metabolism	16	0.01747381	ko00640
Photosynthesis—antenna proteins	15	0.000156573	ko00196
Selenocompound metabolism	11	0.01677999	ko00450

#### Carbon metabolism involved in the response to salt stress in cotton leaves

**Fig 4:**
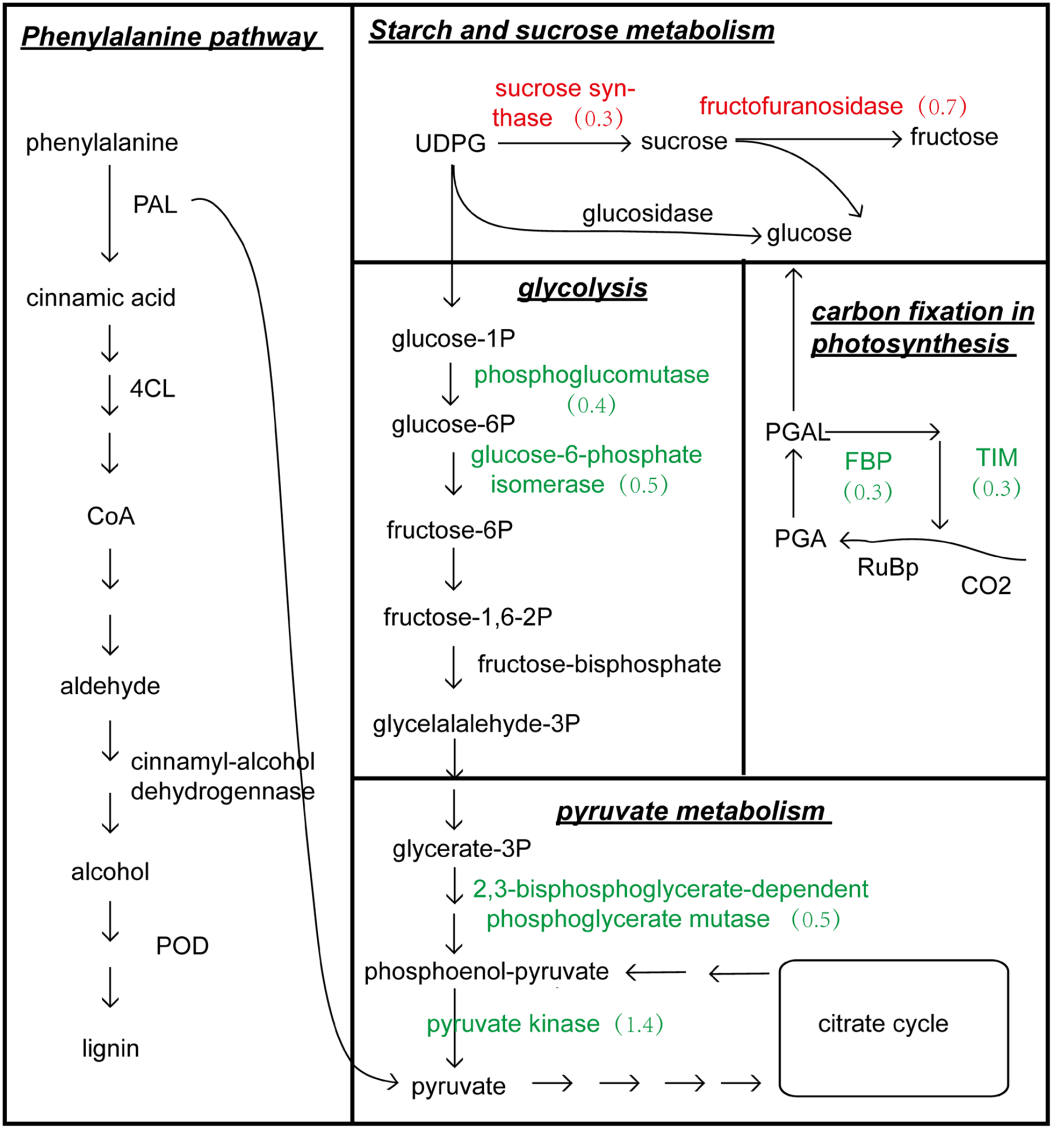
Metabolism pathways involved in the response to salt stress in cotton. Proteins involved in phenylalanine and starch and sucrose metabolism were mostly up-regulated in roots; Proteins involved in photosynthesis, carbon fixation, glycolysis, pyruvate metabolism and phenylalanine were mostly down-regulated in leaves. Red and green numbers represent expression level of up- and down-regulated proteins, respectively. PAL: phenylalanine ammonialyase; 4CL: 4-coumarate—CoA ligase; POD: peroxidase; UDPG: uridine diphosphate glucose; PGAL: glyceraldehyde 3-phosphate; PGA: 3-glyceric acid phosphate.

According to our proteomic profile, carbon metabolism has important roles in response to salinity, particularly with regard to the contributions made by respiration and photosynthesis. The DEPs were conducted with an enrichment analysis and annotated in certain GO categories and KEGG pathways, respectively. The 453 annotated proteins were strongly enriched in GO categories “photosynthesis” (P = 3.06E-10), “photosynthesis, light reaction” (P = 7.09E-05), “electron transport chain” (P = 5.47E-07), “photosynthesis, light harvesting” (P = 3.20E-05), “oxidation-reduction process” (P = 0.00030) and “photosynthetic electron transport chain” (P = 0.00031). The 170 annotated proteins were significantly enriched in pathways of “photosynthesis” (P = 4.39E-13), “Pyruvate metabolism” (P = 0.0018), “Glycolysis / Gluconeogenesis” (P = 0.0116) and “Carbon fixation in photosynthetic organisms” (P = 0.0024). The chlorophyll and carotenoid contents were reduced. Simultaneously, the net photosynthetic rate (P_n_) and stomatal conductance (gs) were inhibited by salinity due to NaCl in cotton variety CCRI-79 [29]. Consistent with this finding, 72 (7.7%) DEPs were enriched in the GO category “photosynthesis”. Among these proteins, 23 were down-regulated and enriched in the same pathway category, including chloroplast proteins in PSII (PsbA-E) and PSI (PsaA, PsaB, PsaF, PsaG, PsaL and PsaN), rieske fes protein (PetA and PetD) in cytochrome b6/f complex, F-type H+-transporting ATPase subunit b. The protein level of two light-harvesting proteins PSI PsaB and PSII PsbE were decreased by 0.4 to 1.2-fold in leaves (Fig 4, S3 Table). A number of proteins involved in “carbon fixation in photosynthetic organism” were down-regulated in leaves, including phosphoenolpyruvate carboxylase (PEPC), malate dehydrogenase, triosephosphate isomerase (TIM), fructose-1, 6-bisphosphatase I (FBP), transketolase (tak) and phosphoglycerate kinase. The protein level were decreased by 0.3 to 1.1-fold (Fig 4, S3 Table). This finding indicated that the photosynthetic capacity were inhibited under salt stress through the involvement of photosynthesis and carbon fixation.

In the process of carbon metabolism, glycolysis and pyruvate metabolism were important as the part of respiration. Most of DEPs were enriched in the pathway category “glycolysis / gluconeogenesis”, including 2, 3-bisphosphoglycerate-dependent phosphoglycerate mutase, phosphoglucomutase and fructose-bisphosphate aldolase. Three protein level was decreased by 0.4 to 0.5-fold. Pyruvate kinase was enriched in the pathway category “pyruvate metabolism”, which was decreased by 1.4-fold (Fig 4, S3 Table). It was concluded that respiration were inhabited to reduce energy consumption in cotton leaves.

#### Starch and sucrose metabolism involved in the response to salt stress in roots

Many proteins related to starch and sucrose metabolism were altered in response to increased salinity in cotton roots. Consistent with this finding, a number of proteins involved in starch and sucrose metabolism were up-regulated in roots (Fig 4, S3 Table). Such proteins included beta-fructofuranosidase, phosphoglucomutase (pgm), pectinesterase, polygalacturonase, trehalose 6-phosphate synthase (otsA), trehalose 6-phosphate phosphatase (otsB), beta-amylase, alpha-amylase and sucrose synthase. The protein level was increased by 0.2- to 0.7-fold (Fig 4, S3 Table). This finding indicated that the carbohydrate biosynthesis and metabolism was possibly developed for energy homeostasis to cope with salt stress in cotton roots.

#### The phenylalanine metabolism involved in the response to salt stress both in roots and leaves

The phenylpropanoid pathway, which includes phenylalanine metabolism and phenylpropanoid biosynthesis, is involved in the stress response of plant cells. On the basis of the proteomic profile, in roots, PAL and 4-coumarate-CoA ligase (4CL) were up-regulated by 1.2 and 0.5-fold, respectively. In leaves, PAL and 4CL were down-regulated by 0.7 and 0.8-fold, respectively (S3 Table). Thus we concluded that phenylalanine metabolism was inhabited and active under salt stress in roots and leaves, respectively.

## Discussion

To cope with salt stress, cotton plants have evolved complex salt-responsive signaling and metabolic processes at the cellular, organ, and whole-plant levels. Concomitant analysis of proteomics results from both the seedling roots and leaves of soybean has facilitated our understanding of how this plant responds to salt stress at the whole-plant level[42]. Several transcriptomic and proteomic analyses of the plant response to salinity have been performed [35,36,43]. In our study, we examined the molecular responses of cotton root and leaves to salt stress by iTRAQ technology. Our proteomics approach has provided a systematic comparison between the salt-responsive metabolic pathways found in cotton seedling leaves and roots under salt stress.

The growth rates of cotton plant roots and leaves decreases with increasing salt concentration [44,45], which may be result in osmotic injury or specific ion toxicity [46]. Compared with the no-salt control treatment, salt stress significantly reduces the growth rates, surface area, volume, and average diameter of the cotton roots, and the dry weights of roots and leaves in cotton variety CCRI-79 [45]. This is accompanied by strong changes in carbohydrate metabolism owing to severe impairments in the photosynthetic and respiration apparatus [47]. In the present study, we found evidence that leaf photosynthetic capacity was weakened under salt conditions, which indicates that this process is sacrificed during salt adaptation in order to conserve energy that then can be redirected to maintaining leaf growth [48]. This was underscored by the down-regulation of chloroplast proteins, an important enzyme involved in the first major photosynthetic step of photosynthesis.

Consistent with this observation, the DEPs were significantly enriched in the photosynthesis pathway. In light reaction. PSI, PSII, cytochrome b6/f and F-type H+-transporting ATP synthase subunits are involved. Photosynthesis takes place in the chloroplasts and the Chl content is important in the regulation and accumulation of PSI and PSII [49,50].

In our previous study, Zhang et al. found that the contents of Chl a, Chl b,and Chl (a+b) were decreased significantly during salinity stress in cotton CCRI-79 [45], which might directly reduce the abundance of these proteins and affect the accumulation of the chloroplast-targeted proteins. In our research, most of PSI and PSII proteins were down-regulated by 0.4- to 1.5-fold. It is reported that photosynthetic proteins were dynamically down-accumulation in mutant defected in Chl biosynthesis [51].

Respiration is an important metabolic pathway that is involved in carbohydrate metabolism in virtually all living organisms. The central role of glycolysis in plants is to provide energy in the form of ATP and to generate precursors such as fatty acids and amino acids for anabolism [48,52]. In this study, Glycolysis and pyruvate pathway were inhabited in leaves, accompanying 2, 3-bisphosphoglycerate-dependent phosphoglycerate mutase, phosphoglucomutase, fructose-bisphosphate aldolase and pyruvate kinase were down-regulated. Our results showed was consistent with the suggestion that increased respiration favors growth maintenance and adaptation to salt stress[48]. Together, these observations led us to conclude that increased energy production via glycolysis and pyruvate metabolism, together with a decreased photosynthetic capacity, maintains energy homeostasis. This adaptive balance permits normal growth of cotton plants even in the presence of salt stress.

Engagement of the phenylpropanoid pathway is one of the critical adaptations that allow plants to survive under stress conditions [53]. Phenylpropanoids are precursors to a wide range of phenolic compounds with many functions in plants. Lignin is a complex phenolic polymer that imparts strength, rigidity, and hydrophobicity to plant secondary cell walls[54]; its formation and subsequent deposition on the cell wall can affect the mechanical strength of supportive and water-conducting tissues[55]. In order to with stand various biotic and abiotic stresses, plants can trigger the production of lignin at specific sites [54,56]. Salt treatment induces root lignification, and increases the number of lignified vessels [57,58]. Lignin monomers are synthesized from phenylalanine[59]. PAL plays an essential role in the phenylpropanoid pathway and is responsive to both biotic and abiotic stress, including pathogen attack, wounding, cold, and UV light [60,61]. PAL catalyzes the first committed step of phenylpropanoid biosynthesis (the deamination of phenylalanine to cinnamic acid) and, along with 4CL, is essential for the synthesis of all phenylpropanoids [59].

Although LC-MS/MS techniques are instrumental in characterizing the peptides, quantitative peptides based biomarker discovery still remains challenging due to the several technological limitations. Among the emerging quantitative technologies, iTRAQ allows the concurrent protein sequence identification and relative quantification of those peptides with known protein sequences in up to eight different biological samples in single experiments [62]. However, due to its limited throughput and current cost, iTRAQ is not feasible to simultaneously compare large sample sizes of salinity subjects to achieve the discovery of differential features of sufficient statistical power [63]. In addition, the success of iTRAQ efforts depends on the peptide sequence determination. Despite our increased understanding of the peptide composition, peptide sequence characterization by the cotton AD genome annotation database (81147 sequences) and the National Center for Biotechnology Information (NCBI) non-redundant fasta database (6833826 sequences) combined, could only determine a small portion peptide features revealed by the HPLC or CE coupled mass spectrometric analysis, which leads to undersampling and incomplete analytic coverage of the peptide. Moreover, it is difficult to meet with the following objectives: to reduce background interference and to increase the signal-to-noise ratio [64]. Therefore, it is necessary to address clearly the limits of iTRAQ and the sources of departure from the theoretical values before using, in order to take full advantage of iTRAQ’s capability in discovering important biomarkers for salt tolerant cotton breeding in the future.

## Conclusions

A proteomic analysis by iTRAQ technology was performed to analyze differential protein expression under salt stress in cotton roots and leaves. In total, 611 and 1477 differentially expressed proteins were identified in roots and leaves, respectively. On the basis of GO and pathway enrichment analysis, we concluded that the “phenylalanine metabolism” and “starch and sucrose metabolism” were active for energy homeostasis to cope with salt stress in cotton roots. Moreover, “photosynthesis”, “pyruvate metabolism”, “glycolysis / Gluconeogenesis”, “carbon fixation in photosynthetic organisms”, and “phenylalanine metabolism” were inhabited to reduce energy consumption in cotton leaves. Further studies for gene function analysis were needed to further clarify the molecular mechanism under salt stress in cotton roots and leaves.

## Ethical standards

The authors note that this research is performed and reported in accordance with ethical standards of the scientific conduct.

## Supporting Information

S1 Table Differentially expressed proteins identified in roots and leaves. (XLSX)

S2 Table The lists of DEPs in GO enrichment analysis. (XLSX)

S3 Table The expression level of DEPs in metabolism pathway. (XLSX)
